# Intense exercise training induces adaptation in expression and responsiveness of cardiac β-adrenoceptors in diabetic rats

**DOI:** 10.1186/1475-2840-9-72

**Published:** 2010-11-05

**Authors:** Solène Le Douairon Lahaye, Arlette Gratas-Delamarche, Ludivine Malardé, Sophie Vincent, Mohamed Sami Zguira, Sophie Lemoine Morel, Paul Delamarche, Hassane Zouhal, François Carré, Françoise Rannou Bekono

**Affiliations:** 1 Laboratory « Movement Sport and health Sciences », UFR APS, University of Rennes 2, Rennes, France; 2 INSERM, U642; University Rennes 1, LTSI; INSERM - CIC-IT 804; CHU Rennes, Department of Biology and Sports Medicine service of functional explorations, Rennes, France

## Abstract

**Background:**

Informations about the effects of intense exercise training on diabetes-induced myocardial dysfunctions are lacking. We have examined the effects of intense exercise training on the cardiac function of diabetic rats, especially focusing on the Langendorff β-adrenergic responsiveness and on the β-adrenoceptors protein expression.

**Methods:**

Control or Streptozotocin induced-diabetic male Wistar rats were randomly assigned to sedentary or trained groups. The training program consisted of 8 weeks running on a treadmill (10° incline, up to 25 m/min, 60 min/day) and was considered to be intense for diabetic rats.

**Results:**

This intense exercise training amplified the *in vivo *diabetes-induced bradycardia. It had no effect on Langendorff basal cardiac contraction and relaxation performances in control and diabetic rats. In diabetic rats, it accentuated the Langendorff reduced responsiveness to β-adrenergic stimulation. It did not blunt the diabetes-induced decrease of β1-adrenoceptors protein expression, displayed a significant decrease in the β2-adrenoceptors protein expression and normalized the β3-adrenoceptors protein expression.

**Conclusions:**

Intense exercise training accentuated the decrease in the myocardial responsiveness to β-adrenergic stimulation induced by diabetes. This defect stems principally from the β2-adrenoceptors protein expression reduction. Thus, these results demonstrate that intense exercise training induces specific effects on the β-adrenergic system in diabetes.

## Background

Diabetes is an increasing health problem worldwide. Independently of coronary vascular diseases, most diabetic patients develop a specific cardiomyopathy characterized by bradycardia and early asymptomatic left ventricular diastolic dysfunction, followed by late systolic dysfunction [1].

In diabetes, the well-known chronic activation of the sympathetic nervous system plays a major role in the cardiac dysfunction by altering expression and/or function of β-adrenoceptors. Previous studies found a redistribution of the β-adrenoceptors subtypes: β1- and β2-adrenoceptors are down-regulated whereas the β3-adrenoceptors are up-regulated in experimental models of diabetes [2,3]. Consequently, the positive inotropic effect of β1- and β2-adrenoceptors is decreased whereas the negative inotropic effect of β3-adrenoceptors is increased [4]. Therefore, this adrenoceptors redistribution likely contributes for a great part to the concomitant impaired contractile responsiveness to β-adrenergic stimulation observed in diabetic hearts [3,5,6]. Moreover, this redistribution might explain in part the well known diabetes-induced bradycardia since the positive chronotropic effect of β1- and β2-adrenoceptors is also decreased. The β3-adrenoceptors influence in this alteration remains less clear because their chronotropic effect is more controversial [7].

Along with insulin, moderate exercise has long been considered to be a major component in the clinical management of type 1 diabetes. Indeed, for its ability to delay the progression of diabetic complications, such as cardiomyopathy [8-11], moderate exercise training is recommended to diabetic patients by the medical community. Scarce data from *in vivo *animal experiments and isolated perfused heart studies found that endurance training improves cardiac output, contractility and relaxation [12-16]. Using experimental diabetes, Bidasee and al. [3] showed that 3 weeks of moderate exercise training, initiated after the onset of diabetes, minimized *in vivo *basal cardiac function loss and improved the cardiac contractile responsiveness to β-adrenergic stimulation. Moderate exercise training also blunted loss of β1-adrenoceptors expression. Interestingly, this moderate exercise training failed to reverse the *in vivo *diabetes-induced bradycardia, did not limit diabetes-induced reduction in β2-adrenoceptors or the increase of β3-adrenoceptors expression and only minimally improved cardiac output. The reason for the lack of training effect on β2- and β3-adrenoceptors expression is not clear at this time. As suggested by the authors, the load of exercise training (duration and perhaps intensity) was not sufficient enough to induce significant effects. Information about the effect of more intense or prolonged exercise training on cardiac function or on β-adrenergic system in diabetes is scarce. In a preliminary study [17], we demonstrated that 8 weeks of intense exercise training has specific and opposite effects to those obtained with more moderate exercise training [3] on β2-adrenoceptors expression in diabetic rat hearts. Intense exercise training during diabetes decreases cardiac β2-adrenoceptors expression. Thus, based on these data and others in different experimental models (healthy rats, obese rabbits...), we hypothesized that exercise training may affect differently all of the above mentioned factors depending on the load [18,19]. The present study was conducted to investigate the effect of 8 weeks of intense exercise training on both cardiac function and β-adrenergic system in diabetic rat hearts.

## Methods

### Chemicals and drugs

Streptozotocin (STZ) and Isoproterenol Hydrochrloride were purchased from Sigma-Aldrich Chemical (France). Rabbit polyclonal anti-β1-adrenoceptors and anti-β2-adrenoceptors, goat polyclonal anti-β3-adrenoceptors and mouse monoclonal anti-HSC 70 were all purchased from Tebu-Bio International (France). Horseradish peroxydase (HRP)-conjugated IgG polyclonal donkey anti-goat was from Tebu-Bio International (France). Goat anti-rabbit and goat anti-mouse secondary antobodies were purchased from Dako Cytomation (France).

### Experimental models

All the procedures were approved by the Institutional Animal Care and Use Committee of the University of Rennes (France) and were carried out following the use of the French Farming Minister and the Guide for the Care and Use of the Laboratory Animals. The study was conducted in male Wistar rats housed in an animal room on an inverse 12:12-h light-dark cycle and given access to water and food *ad libitum *throughout the duration of the study. The rats (*n *= 125; from Janvier, France), 9 weeks old at the beginning of the experiment (350 g), were randomly assigned into 4 groups, a sedentary control (SC), a trained control (TC), a sedentary diabetic (SD), and a trained diabetic (TD) groups. In each group, a minimum of 7 rats were used for the Langendorff isolated heart study on the one hand and a minimum of 7 other rats for the Western blot analysis on the other hand. 8 rats were not included in the experiment for 2 different reasons: death or refusal of treadmill running. The final number of rats for this study was 117.

### Induction of experimental streptozotocin-induced diabetes

Animals were injected with either an intraperitoneal single dose of STZ in 0.1 M citrate buffer, pH 4.5 (45 mg.kg^-1^) (SD, TD) or citrate buffer only (SC, TC). Three days later, blood glucose levels were determined using a glucometer (MediSense Optium). The onset of diabetes was determined by blood glucose concentration > 250 mg.dL^-1 ^[20]. The detection of ketones using the glucometer or urinary strips (Keto-Diastix^®^-Bayer Diagnostic) and body weight loss confirm diabetes.

### Training protocol

One week after the STZ or citrate buffer injection the animals were exercised. Physical training consisted of progressive running up to 25 m/min, 60 minutes, 5 days/week for 8 weeks on a rodent treadmill (Exer 3/6 Treadmill, Columbus Instruments) set at incline 10°, as previously used [17]. For the first 2 weeks, each exercise bout consisted of 10 min of running at 20 m/min. The following 3 weeks consisted progressively of 40 min at 22 m/min. For the remaining 3 weeks of the protocol, the duration was increased to 60 min at 25 m/min. Based on the studies of Le Douairon Lahaye and al. [17] and Rodrigues et al. [21] we can assume that for a same running speed, diabetic rats used a higher percentage of their VO_2 _max than control ones and consequently that this is an intense training program for diabetic rats. Only animals which ran steadily on the treadmill were included in the study.

All rats were sacrificed 24 h after the last session of training.

### Citrate synthase activity

Frozen gastrocnemius tissue (200 mg) was used to assay for citrate synthase (CS). Muscle samples were homogenized (1/10 w/v) in a buffer solution pH 7.5 containing Na_2_HPO_4 _(0.1 mol.L^-1^), NaH_2_PO_4_, H_2_O (0.1 mol.L^-1^), and EDTA (2 mmol.L^-1^) for 20 s at 30000 rpm with a polytron. The homogenate was then sonicated 6 × 10 s and centrifuged at 1500 G for 13 min at 4°C in duplicate. Citrate synthase activity was spectrophotometrically determined in duplicate in protein extracts at 25°C as described previously [22] with few modifications. Results were expressed in μmol.ml^-1^.min^-1^.g tissue^-1 ^for each group.

### *In vivo *heart rate recordings

*In vivo *baseline heart rate (HR) of rats was measured using the tail cuff method (Phymep, France). After habituation of animals, heart rate was measured twice, at the onset of protocol (T1) and before their sacrifice (T2). All measurements were made at the same time to accommodate for diurnal variations.

### Isolated rat heart preparation

Rats were anesthetized with sodium pentobarbital (50 mg.kg^-1^, i.p) and heparinized (500 U.kg^-1^, i.p). Isolated rat hearts were immediately attached to the Langendorff perfusion apparatus (Phymep, France) and retrogradely perfused (Peristaltique pump, Gibson, Paris) at a constant perfusion pressure with oxygenated Krebs-Henseleit buffer containing NaCl (118 mM), KCl (4.7 mM), CaCl_2 _(1.9 mM), MgSO_4 _(1.2 mM), KH_2_PO_4 _(1.2 mM), NaHCO_3 _(25 mM), glucose (11 mM), pH7.4 [23]. The perfusate was equilibrated with a standard 95% O_2_/5% CO_2 _gas mixture and maintained at 37°C. A balloon was inserted into the left ventricular (LV) cavity, and its volume was adjusted to 10-15 mmHg of LV end-diatolic pressure (LVEDP). All hearts were placed in a thermostatically controlled room (37°C). After equilibration, pre-agonist baseline data was recorded (PowerLab, ADInstruments). After baseline, 5-minute infusions of isoproterenol were begun at doses ranging from 1.10^-8 ^to 1.10^-5 ^M. Each isoproterenol infusion was stopped after 5 minutes, and hearts were allowed to return to baseline before the next dose was initiated, as described previously [24]. In trained control rats, 1.10^-6 ^and 1.10^-5 ^M isoproterenol doses were not tested for technical reason. LV systolic pressure (LVSP), LVEDP, and the maximum rate of positive and negative change in LV pressure (± dP/dt) were continuously recorded. LV developed pressure (LVDP) was calculated by subtracting the LVEDP from LVSP. Rate-pressure product (RPP = heart rate × LVDP) was also calculated as an index of cardiac performance.

### Protein assay

About 200 mg of LV samples were homogenized in a homogenization buffer (HB) containing (in mM) Tris 20 (pH 7.4), NaCl 20, MgCl_2 _5 and protease inhibitor cocktail (from Roche Molecular Biochemicals, Roche Diagnostics, France). The homogenates were then centrifuged at 500 G for 5 min at 4°C. Supernatants were recovered and centrifuged at 30.000 G for 30 min at 4°C. Pellets were resuspended in HB. The homogenates were then aliquoted and stored at -80°C. The total protein concentration was determined using the Lowry method [25]. Chemicals were purchased from Sigma Aldrich (France).

### Western blot analysis

Western blot analyses were used to determine relative levels of β1-, β2-, and β3-adrenoceptors. Briefly, samples were solubilized in buffer containing Tris-HCl (pH 6.8), SDS, bromophenol blue, glycerol and 2-β-mercaptoethanol. Proteins were separated on a SDS-polyacrylamide gel electrophoresis (10%) and then transferred overnight into PVDF membranes (Millipores) in a transfer buffer (25 mM Tris, 192 mM Glycine, 0.01% SDS and 10% ethanol). For normalisation, a same protein sample was deposed on each gel. After transfer, the membrane was washed in Phosphate-buffered saline/Tween 20 (TPBS 0.1%) for 10 min. Transfer was checked by staining of the blots in Ponceau S solution. After blocking nonspecific binding sites for 2 h at room temperature by 5% non-fat milk diluted in TPBS 0.1%, membranes were incubated for 2 h at room temperature with either anti β1-, β2-, or β3-adrenoceptor antibodies. Membranes were washed three times with TPBS and then incubated for 1 h with either anti-rabbit IgG-horseradish peroxidase (β1-and β2-adrenoceptors) or anti-goat IgG-horseradish peroxidase (β3-adrenoceptors). Finally, immunoreactive bands were visualized by chemiluminescence and quantified by densitometry using a computer-based imaging system (MultiGauge Fujifilm, France). Western blots were normalized to house-keeping protein (HSC 70: constitutive isoform of heat shock protein 70) and expressed in percentage of control values.

### Data analysis

Animal characteristics and myocardial protein expression were compared by use of 1-way ANOVA, followed by a Fisher LSD post-hoc analysis. The temporal responses of HR and functional cardiac parameters to training were analyzed with ANOVA for repeated measures and Fisher LSD post-hoc analysis. Isoproterenol dose-response relationships were compared by ANOVA for repeated measures, followed by 1-way ANOVA and Fisher LSD post-hoc analysis at each isoproterenol concentration. All analyses were performed on Statistica 7.1 (Statsoft, France). A level of *p *<*0.05 *was selected to indicate statistical significance. All values were expressed as mean ± SEM.

## Results

### Animal characteristics

The general characteristics of the rats at time of sacrifice are reported in table 1. As expected, the level of blood glucose was much higher in sedentary diabetic rats than in control rats (*p *<*0.001*). Training reduced the levels of blood glucose, which was lower in trained diabetic rats than in their sedentary counterparts (*p *<*0.001*). At the end of the study, body weights were significantly reduced in both sedentary and trained diabetic rats, relative to sedentary control rats (*p *<*0.001*). Sedentary control rats had significantly higher body weights than trained control rats (*p *<*0.05*), whereas sedentary diabetic rats had significantly lower body weights than trained diabetic rats (*p *<*0.05*). Similarly, diabetes induced a pronounced reduction in the left ventricular weight of both sedentary and trained rats, relative to controls (*p *<*0.001*). With regard to left ventricular muscle/body weight ratio, no evidence of cardiac hypertrophy was observed following the treadmill training program.

**Table 1:** General characteristics of animals

	Sedentary controls (*n *= 34)	Trained controls (*n *= 20)	Sedentary diabetics (*n *= 35)	Trained diabetics (*n *= 28)
BW (g)	504.1 ± 6.3	478.3 ± 10.3 *	293.1 ± 11.4 *†	330.2 ± 11.4 *†‡
HW (mg)	1263.5 ± 31.3	1244.3 ± 40.7	861.8 ± 46.8*†	946.7 ± 34.5*
LVW (mg)	1106.0 ± 42.1	1104.8 ± 48.7	741.7 ± 41.1*†	878.6 ± 53.5*†‡
HW/BW (mg/g)	2.5 ± 0.1	2.7 ± 0.1	3.4 ± 0.1*	2.9 ± 0.1*‡
LVW/BW (mg/g)	2.2 ± 0.1	2.3 ± 0.3	2.6 ± 0.1*†	2.6 ± 0.1*†
Blood glucose (mg/dl)	128.7 ± 3.7	124.3 ± 3.7	556.2 ± 13.6*†	497.4 ± 23.8*†‡

BW indicates body weight; HW, heart weight; LVW, left ventricular weight; HW/BW, heart/body weight; LVW/BW, left ventricular/body weight. Data are presented as mean ± SEM. *= significantly different from sedentary control rats (*p *<*0.05*), † = significantly different from trained control rats, ‡ = significantly different sedentary diabetic rats (*p *<*0.05*).

### Exercise training induces a higher increase in citrate synthase activity in diabetic rats than in control rats

To determine the training program effectiveness, we measured the CS activity, an index of skeletal muscle oxidative capacity. Gastrocnemius CS activity is significantly increased in trained rats compared with their respective sedentary counterparts (TD, 100 ± 9 vs SD 61 ± 6 (*p *<*0.005*), and TC, 69 ± 4 vs SC, 56 ± 1 (*p *<*0.05*) μmol.mL^-1^.min^-1^.g tissue^-1^). This increase of CS activity leads to the conclusion that the training program is efficient. Moreover, trained diabetic animals exhibited a higher increase in CS activity than trained control animals (TD, +56% vs TC, +24%). These results suggest that the training program used in the present study required more effort (was a more intense exercise program) for the diabetic rats than the normal rats, certainly because diabetes severely depresses physical fitness [21,26]. Moreover, in this study, the increase in CS activity in trained diabetic rats is higher than in diabetic rats exposed to moderate exercise training [3]. Such results lead to defining this training program as intense exercise training for diabetic rats and moderate exercise training for healthy rats.

### In diabetic rats, intense exercise training does not restore basal cardiac function

*In vivo *heart rate - Diabetes induced an *in vivo *bradycardia because sedentary diabetic rats had significant lower *in vivo *HR compared to sedentary control rats (*p *<*0.001*) (Table 2). Exercise training decreased the *in vivo *HR in both control and diabetic rats compared to their respective sedentary counterparts (*p *<*0.01*).

**Table 2:** *In vivo *tail cuff data

	Heart Rate (bpm)	
	**T1**	**T2**

Sedentary controls (*n* = 34)	412 ± 21	387 ± 9
Trained controls (*n *= 20)	423 ± 12	389 ± 6 **¤**
Sedentary diabetics (*n *= 35)	340 ± 9 *†	338 ± 8 *†
Trained diabetics (*n *= 28)	331 ± 8 *†	310 ± 8 *†‡ **¤**

Data are presented as mean ± SEM. ¤ = significant difference between T1 (at the onset of protocol) and T2 (before the sacrifice) (*p *<*0.05*), *= significantly different from sedentary control rats (*p *<*0.05*), † = significantly different from trained control rats (*p *<*0.05*), ‡ = significantly different from sedentary diabetic rats (*p *<*0.05*).

**Figure 1:**
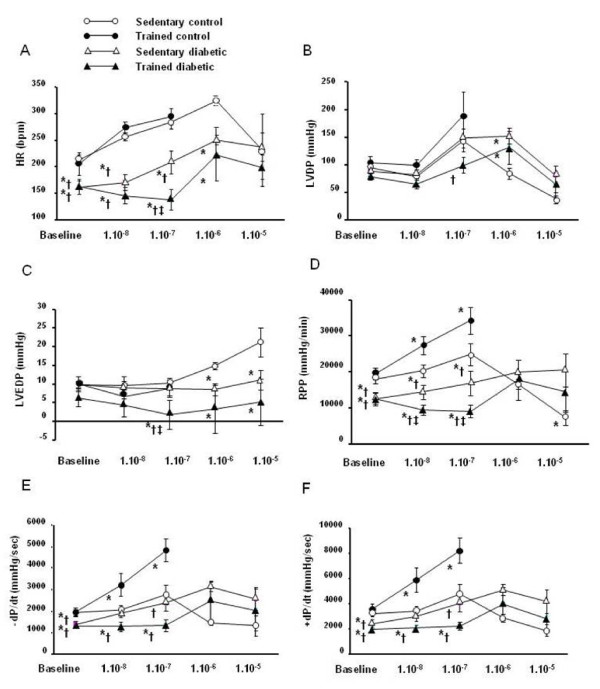
**Effect of intense exercise training on myocardial basal function and responsiveness to isoproterenol stimulation**. Isoproterenol was infused to isolated hearts of sedentary control (*n *= 13), trained control (*n *= 7), sedentary diabetic (*n *= 11) and trained diabetic (*n *= 7) rats in ascending concentrations ranging from 1.10^-8 ^to 1.10^-5 ^M. In trained control rats, 1.10^-6 ^and 1.10^-5 ^M isoproterenol doses were not tested for technical reason. Data are presented as mean ± SEM. *= significantly different from sedentary controls (*p *<*0.05*), † = significantly different from trained controls (*p *<*0.05*), ‡ = significantly different from sedentary diabetics (*p *<*0.05*).

Intrinsic heart rate - Diabetes also induced an intrinsic bradycardia. Intrinsic HR was ≈ 50 bpm lower in sedentary diabetic rats than in sedentary control rats (*p *<*0.05*) (Figure 1A). Exercise training had no significant effect on the intrinsic diabetic bradycardia. Similarly, no significant effect of exercise training was observed in intrinsic HR of control rats.

Intrinsic contraction and relaxation functions - Diabetes caused a decline in cardiac basal function (Figures 1B-F). Whereas LVDP and LVEDP were not significantly altered, the RPP as well as the respective contraction and relaxation indexes (± dP/dt) were significantly lower in sedentary diabetic rats compared to sedentary control rats (*p *<*0.05*). Exercise training had no significant effect on all these parameters whatever the group, control or diabetic.

### In diabetic rats, intense exercise training decreases the myocardial responsiveness to isoproterenol stimulation

Isoproterenol significantly increased HR, LVDP, RPP and ± dP/dt in all groups (Figure 1). Nevertheless, diabetes induced a rightward shift in the isoproterenol dose-response relationships without altering the magnitude of the peak responses (Figure 1). Indeed, if the threshold responses of sedentary diabetic rat hearts were obtained for 1.10^-7 ^M dose as sedentary control rats, their peak responses were obtained for 1.10^-6 ^M dose. Exercise training amplified the rightward shift in diabetic rats whereas it induced a leftward shift in control rats. Indeed, in trained diabetic rats, 1.10^-7 ^M dose had no effect on HR, LVDP, LVEDP, RPP as well as the respective contraction and relaxation indexes. 1.10^-6 ^M dose was needed to trigger a significant response. In contrast, in trained control rats, 1.10^-8 ^M dose was sufficient to induce a significant response.

### In diabetic rats, intense exercise training decreases cardiac β2-adrenoceptors and normalizes β3-adrenoceptors expression

**Figure 2:**
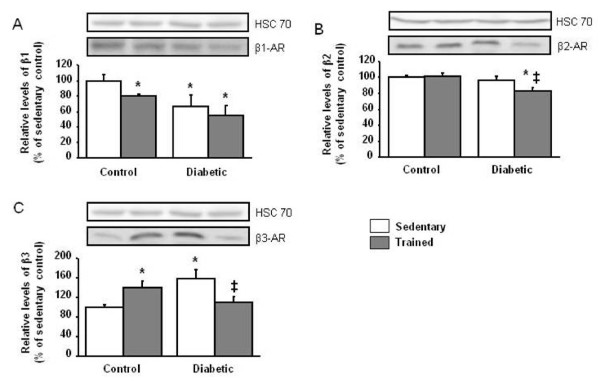
**Effect of intense exercise training on β-adrenoceptors expression**. Representative Western blots show steady state levels of β1-, β2- and β3-adrenoceptors in left ventricular muscles from sedentary control (*n *= 8), trained control (*n *= 8), sedentary diabetic (*n *= 7) and trained diabetic (*n *= 9) rat hearts. Data are presented as mean ± SEM. *= significantly different from sedentary controls (*p *<*0.05*), ‡ = significantly different from sedentary diabetics (*p *<*0.05*).

Diabetes was associated with a down-regulation of β1-adrenoceptors (*p *<*0.05*) and an up-regulation of β3-adrenoceptors (*p *<*0.005*), without any change in β2-adrenoceptors expression (Figure 2). For control rats, exercise training decreased the expression of β1-adrenoceptors (*p *<*0.01*) and increased the expression of β3-adrenoceptors (*p *<*0.05*). In diabetic rats, exercise training exerted specific adaptations in β-adrenergic distribution. Whereas it did not restore the β1-adrenoceptors expression, it displayed a significant decrease in the β2-adrenoceptors expression (*p *<*0.01*) and normalized the β3-adrenoceptors expression.

## Discussion

In the present study, a multifaceted approach was used to study for the first time the intense exercise training-induced adaptations of cardiac function focusing on the β-adrenergic system in diabetic rats. The major finding of this study is that intense exercise training accentuated the decrease in the myocardial responsiveness to β-adrenergic stimulation induced by diabetes. This defect stems principally from the β2-adrenoceptors protein expression reduction. Thus, these results demonstrate that intense exercise training induces specific effects on the β-adrenergic system in diabetes.

Diabetes is well-known to induce pathological bradycardia [14]. Responsible mechanisms are complex and multifactorial; likely associating alterations of extrinsic and intrinsic myocardial properties. To discriminate between the two, it is interesting to study the effects of exercise training on the heart rate evaluated *in vivo *and in Langendorff isolated perfused hearts. Whereas exercise training accentuates the *in vivo *bradycardia in diabetic rats, no significant effect is observed in isolated perfused diabetic hearts. Similar results are observed in control rats since they only exhibit a decrease *in vivo *heart rate after exercise training. These findings suggest that exercise training program essentially affects the cardiac extrinsic properties involved in the chronotropic function.

As in many prior studies [27-29], diabetes causes a decline in cardiac basal function. As for the chonotropic function, exercise training does not affect contraction and relaxation function parameters. Indeed, we found that in isolated perfused hearts, the left ventricular developed pressure, the LVEDP, the rate-pressure product and the respective contraction and relaxation indexes (± dP/dt) are not modified by exercise training whatever the group, control or diabetic. These results suggest that exercise training has no effect on the intrinsic function of the heart. It is therefore likely that in both control and diabetic rats exercise training might modify cardiac extrinsic properties such as baroreflex sensitivity [30], vagal tone [14,31], circulating hormonal factors [32], or β-adrenergic function [2,3,18,33].

Literature data concerning the effects of exercise training on cardiac β-adrenergic function remains non univocal depending on the subject (healthy or pathological) or on the exercise load (moderate or intense) [3,19,33]. As for obese rabbits [19], we hypothesized that intense exercise training in diabetic rats might decrease myocardial responsiveness to β-adrenergic stimulation. Consistent with an earlier study [3], we found that diabetes induces a rightward shift in the isoproterenol dose-response relationships without altering the magnitude of the peak responses. Exercise training amplifies the rightward shift in diabetic rats whereas it induces a leftward shift in control rats. Thus, as previously described [34], we found that exercise training improves the β-adrenergic responsiveness in healthy rat hearts. However, the major novel is that this intense exercise training for diabetic rats aggravates the diabetes-induced reduction of myocardial β-adrenergic responsiveness. These data highlight the fact that exercise training affects differently the myocardial responsiveness to β-adrenergic stimulation depending on the exercise load. Indeed, moderate exercise training in diabetic rats has been found to also increase the *in vivo *responsiveness to β-adrenergic stimulation [3]. In the case of obese rabbits, moderate endurance training failed to attenuate obesity-related decrements in myocardial responsiveness whereas intense endurance training caused reduced responsiveness to isoproterenol stimulation [19].

Since it is well recognized that β-adrenoceptors redistribution is a key factor able to influence the myocardial β-adrenergic responsiveness, we further investigated the myocardial expression of the three β-adrenoceptors subtypes after exercise training. As previously described [2,3,17], diabetes is associated with a down-regulation of β1-adrenoceptors and an up-regulation of β3-adrenoceptors, without any change in β2-adrenoceptors expression. For control rats, exercise training decreases the expression of β1-adrenoceptors and increases the expression of β3-adrenoceptors. Thus, to some extent, exercise training induces similar adaptations in β-adrenoceptors expression as does diabetes. These results have been previously described [18] but underlying responsible mechanisms still need clarification. The present study is the first to analyze the effects of intense exercise training in diabetic rats. We found that intense exercise training exerts specific adaptations in β-adrenergic function. In a preliminary study [17], we demonstrated that intense exercise training exerts specific adaptations in β-adrenergic distribution. These results are confirmed in the present study. While exercise training does not restore the β1-adrenoceptors expression, it produced significant decreases in the β2-adrenoceptors expression and normalized the β3-adrenoceptors expression in diabetic rats. Here also, opposite results have been found in diabetic rats exposed to moderate exercise training which restores the β1-adrenoceptors expression without affecting the diabetes-induced β2- and β3-adrenoceptors redistribution [3]. These differences could probably account for a higher activation of the sympathetic nervous system. Indeed, the exercise training program used in this study is certainly generating an important stress, higher than that used by Bidasee et al. [3]. This important stress might induce a significant increase in myocardial catecholamine levels as suggested by Zouhal et al. [35]. Thus, in diabetic rats, the stress induced by intense exercise training could be added to the stress induced by diabetes. Less sensitive to catecholamines than the β1-adrenoceptors, the β2-adrenoceptors would be negatively regulated under the effect of this double stress [36].

This study demonstrates for the first time that in diabetes, intense exercise training induces specific effects on the β-adrenergic system. These specific effects are characterized by an aggravation of the reduced myocardial β-adrenergic responsiveness induced by diabetes and a particular redistribution of β-adrenoceptors, more specifically the appearance of a reduction in the β2-adrenoceptors expression and a normalization of β3-adrenoceptors expression. The corrective effect of exercise training on β3-adrenoceptors expression in diabetic rats and its functional consequences remain to be clarified. To date, the β3-adrenoceptors-mediated signalling pathways are less documented and further investigations will be required to define the effects of intense exercise training on diabetic cardiac functions linked to β3-adrenoceptors.

Nevertheless, our results strongly suggest that the intense exercise training-induced accentuation of the reduced myocardial β-adrenergic responsiveness in diabetic rats stems principally from the reduction in β2-adrenoceptors expression.

Only hypotheses might be advanced to determine the clinical implications of these specific adaptations. As the β2-adrenoceptors stimulation has been demonstrated to be arrhythmogenic in both failing human ventricular cardiomyocytes and failing canine myocytes [37,38] as well in rabbit heart failure myocytes [39], we hypothesized that the effects of exercise training on the β2-adrenoceptors function in diabetic rat hearts might be protective. In dogs with elevated susceptibility to ventricular fibrillation, Billman et al. [40] and Hollycross et al. [41] found that intense exercise training attenuates myocardial responsiveness to β2-adrenergic stimulation. The authors suggested that this specific adaptation prevented ventricular fibrillation. Future studies should be planned to investigate the specific effects of intense exercise training on the β2-adrenergic responsiveness in diabetic rat hearts and to evaluate the clinical implications.

### Study Limitations

The interpretability of our study is limited in several ways. First, the 1.10^-7 ^M isoproterenol dose was most likely extreme for trained control rats. This dose elicited an irreversible effect on myocardial function of this group which failed to test the next dose. Thus, due to this technical difficulty, we were not able to study the response of trained control rat heart to 1.10^-6 ^and 1.10^-5 ^M doses as in diabetic ones. The reason for this effect is not clear at this time; further studies are required to elucidate this point. Second, we did not examine myocardial microcirculation. However, pulmonary hypertension and myocardial blood flow reserve alteration seem to be important factors in myocardial disease in diabetes [42,43]. It has been demonstrated that diabetes induces a functional alteration of the myocardial microcirculation that may explain the left ventricular systolic dysfunction observed in diabetic animals [42]. Thus, it will be interesting to study the effects of exercise training on the myocardial microcirculation and the consequences on the cardiac function of diabetic rats.

## Conclusions

In conclusion, this study demonstrates that intense exercise training induces specific effects on the β-adrenergic system in diabetic rat hearts. Intense exercise training decreases the myocardial responsiveness to isoproterenol stimulation and produces a decrease in cardiac β2-adrenoceptors expression. The clinical implications will however need to be clarified although we can assume that these specific adaptations could protect the diabetic heart against the risk of arrhythmia during intense exercise training.

## Competing interests

The authors declare that they have no competing interests.

## Authors' contributions

SLDL was responsible for the design conception of the experiments, collection, analysis and interpretation of the data, and drafting of the manuscript. AGD was responsible for the interpretation of the data and drafting of the manuscript. LM, SV, MSZ, SLM, and HZ were responsible for exercise training protocol. PD supervised the study. FC helped to interpret the data and to draft the manuscript. FRB was responsible for the design conception of the experiments, interpretation of the data, and drafting of the manuscript. All authors have read and approved the final manuscript.
